# Detection and sequence/structure mapping of biophysical constraints to protein variation in saturated mutational libraries and protein sequence alignments with a dedicated server

**DOI:** 10.1186/s12859-016-1124-4

**Published:** 2016-06-17

**Authors:** Luciano A. Abriata, Christophe Bovigny, Matteo Dal Peraro

**Affiliations:** Laboratory for Biomolecular Modeling, Institute of Bioengineering, School of Life Sciences, École Polytechnique Fédérale de Lausanne, and Swiss Institute of Bioinformatics, AAB014 Station 19, Lausanne, 1015 Switzerland; Present address: Molecular Modeling Group, Swiss Institute of Bioinformatics, UNIL, Bâtiment Génopode, Lausanne, 1015 Switzerland

**Keywords:** Deep sequencing, Next-generation sequencing, High-throughput, Protein evolution, Protein design, structure-function relationships, Protein biophysics, Structural biology, Neutral drift

## Abstract

**Background:**

Protein variability can now be studied by measuring high-resolution tolerance-to-substitution maps and fitness landscapes in saturated mutational libraries. But these rich and expensive datasets are typically interpreted coarsely, restricting detailed analyses to positions of extremely high or low variability or dubbed important beforehand based on existing knowledge about active sites, interaction surfaces, (de)stabilizing mutations, etc.

**Results:**

Our new webserver *PsychoProt* (freely available without registration at http://psychoprot.epfl.ch or at http://lucianoabriata.altervista.org/psychoprot/index.html) helps to detect, quantify, and sequence/structure map the biophysical and biochemical traits that shape amino acid preferences throughout a protein as determined by deep-sequencing of saturated mutational libraries or from large alignments of naturally occurring variants.

**Discussion:**

We exemplify how *PsychoProt* helps to (i) unveil protein structure-function relationships from experiments and from alignments that are consistent with structures according to coevolution analysis, (ii) recall global information about structural and functional features and identify hitherto unknown constraints to variation in alignments, and (iii) point at different sources of variation among related experimental datasets or between experimental and alignment-based data. Remarkably, metabolic costs of the amino acids pose strong constraints to variability at protein surfaces in nature but not in the laboratory. This and other differences call for caution when extrapolating results from in vitro experiments to natural scenarios in, for example, studies of protein evolution.

**Conclusion:**

We show through examples how PsychoProt can be a useful tool for the broad communities of structural biology and molecular evolution, particularly for studies about protein modeling, evolution and design.

**Electronic supplementary material:**

The online version of this article (doi:10.1186/s12859-016-1124-4) contains supplementary material, which is available to authorized users.

## Background

Deep-sequencing technologies are revolutionizing structural and evolutionary biology by (i) providing experimental maps of tolerance to substitutions and fitness landscapes for proteins [1–20] and (ii) by boosting genomic projects thus increasing the coverage of protein families to an extent that even allows for structural modeling through analysis of residue coevolution [21–27].

In deep-sequencing-based measurements of tolerance to substitutions and fitness landscapes, saturated mutational libraries of a protein or segment of interest are selected for a trait and the retained variants are all sequenced at once to quantify the frequency of each amino acid at each position after selection or, in most cases, the change in frequency before and after selection. The datasets resulting from such complex and costly experiments are usually analyzed in terms of the specific questions the selection experiment was designed to answer, looking coarsely at the extent of conservation and variability throughout the subject protein and focusing structural and functional interpretations mainly on residues of exceptionally high or low tolerance to substitutions and on specific residues known to be critical for stability, catalytic activity, molecular binding, etc. However, these datasets are inherently rich in finer information, intrinsically related to how protein sequences can drift, evolve and be engineered. On the other hand, the fact that sequence coverage in protein families is good enough to derive 3D models of proteins and protein complexes from residue coevolution analysis suggests that other kinds of structural/functional information might be available from large structure-consistent alignments.

Here we present *PsychoProt*, a web-based tool to systematically extract quantitative biochemical/biophysical information about site-specific amino acid variability from deep-sequencing experiments of saturated mutational libraries or from protein sequence alignments. Specifically, *PsychoProt* unveils explanations for the amino acid preferences observed throughout a subject protein or segment of interest by finding out which amino acid descriptors shape the probability of observing each amino acid at each site, and maps these constraints on the protein’s sequence and structure. The descriptors, separated in sets that can be independently tested, include physicochemical properties of the amino acids, metrics for their metabolic costs and discrete numbers describing the atomic composition of their side chains.

We begin by describing *PsychoProt*’s main inputs, outputs and internal algorithms, and we then illustrate how to interpret *PsychoProt*’s results through examples where we have analyzed amino acid variation in deep-sequencing datasets of saturated mutational libraries and in alignments of naturally occurring proteins consistent with the proteins’ folds according to coevolution analyses. Five remarkable highlights arise from these examples. First, we uncover several sites under mechanical constraints at the core of a protein that undergoes refolding for its main function. Second, we note different physicochemical constraints acting on two variants of a nucleic acid-binding protein despite their high sequence identity. Third, we uncover extensive constraints on flexibility around the active site of a family of hydrolases with large substrate promiscuity. Fourth, we report different constraints acting on a protein upon *in vitro* experimentation in the laboratory compared to the same protein in a structure-consistent alignment of natural variants, particularly with a large number of surface sites that favor metabolically inexpensive amino acids in the natural variants but not in the experimental dataset. Fifth, we report for protein alignments pervasive constraints for hydrophobicity and hydrophilicity, followed by metabolic descriptors and then by descriptors related to volumes, steric hindrance and flexibility, in patterns that might be exploited to identify structural and functional features of proteins from alignments only.

## Implementation

### Server inputs

*PsychoProt* works entirely online through a graphical interface available for free without registration at http://psychoprot.epfl.ch (or at http://lucianoabriata.altervista.org/psychoprot/index.html). Its input is in principle any dataset describing amino acid variation across a protein sequence or segment of interest, coming either from deep-sequencing of saturated mutational libraries or simply from large sequence alignments. The input must be formatted as a text table containing the preferences for each of the 20 amino acids (the amino acid “distributions”) at all positions (“sites”, or residues) of interest. A metric for the total tolerance to substitution k* for each site can optionally be provided too. The input table can be computed in any spreadsheet program and copied into *Psychoprot*’s input box. See Methods and the examples provided in the website for further details.

Amino acid preferences can in principle consist of any metric that increases monotonically with the probability of observing each amino acid at each position [4, 28–33]. Among such possible metrics, the best one is likely dependent on the exact source of information and experimental setup (the examples taken from the literature and analyzed later on in this work indeed utilized different metrics). As shown in three examples, we found the “statistical free energy” particularly useful to encode data from sequence alignments, as it recovers known aspects of protein chemistry suggesting that new insights will likely be relevant:

1 $$ \varDelta \varDelta {G}_{i,j}\kern0.5em =\kern0.5em -{ \log}_{10}\kern0.5em \left({P}_{i,j}/{P}_{wt,j}\right) $$

where *P*_*i,j*_*/P*_*wt,j*_ is the ratio of pseudocounts of amino acid type *i* relative to the wild type amino acid at position *j*. To process an alignment of protein sequences, an auxiliary online tool converts it into a proper input table containing all the *ΔΔG*_*i,j*_ values. This possibility allows biochemists and structural biologists to easily analyze variation in their protein alignments beyond the simple examinations of conservation that most programs offer.

Last, if the user does not provide any metric for the total tolerance to substitutions at each site, or when the auxiliary tool builds an input table from alignments, *PsychoProt* computes it as:

2 $$ {k}_j^{*}\kern0.5em =\kern0.5em {2}^{\varSigma_{i\kern0.5em =1}^{20}\kern0.5em {P}_{i,j}\kern0.5em { \log}_2\kern0.5em {P}_{i,j}} $$

where the frequencies for each amino acid at each site are calculated from the given preferences, assuming that they are statistical free energies solving for *P*_*i,j*_ from eq. 1 relative to *P*_*i,wt*_ = 1.

### Algorithm and outputs

Given the input, *PsychoProt* scans the amino acid preferences at each site against a set of amino acid descriptors testing for fits of statistically significant correlation considering the Bonferroni correction for multiple testing [34]. For this the user defines a cutoff for the *p*-value associated to the probability that the observed correlation is obtained simply by chance, a strategy that improves upon our previous exploratory study [35]. The list of supported amino acid descriptors (Table 1) is split in Sets, of which the first two are the simplest to interpret and whose descriptors are low-correlated to each other so they are selected by default and recommended for normal use. Set 1 contains eight basic physicochemical descriptors of the amino acids: *volume*, *log(solubility)*, *hydrophobicity*, *isoelectric point*, *helix propensity*, *steric hindrance*, *flexibility* and *sheet propensity.* Set 2 contains two metrics related to the metabolic cost of the amino acids both having larger values for metabolically more expensive amino acids [36].

**Table 1:** List of amino acid descriptors available in *PsychoProt*. Only sets 1 and 2 are recommended for normal use

Set 1	Physicochemical descriptors	Set 3	Composite physicochemical descriptors
	Volume		Volume/P(helix)
	Log(solubility)		Volume/P(sheet)
	Hydrophobicity		Volume/log(solubility)
	Isoelectric point		Log(solubility) x Flexibility
	P(helix)		Steric hindrance x Flexibility
	Steric hindrance		P(helix) + P(sheet)
	P(sheet)		log(solubility) x Hydrophobicity
	Flexibility		Hydrophobicity x Flexibility
Set 2	Metabolic descriptors		Volume/(P(helix) + P(sheet))
	In vivo decay time		Steric hindrance/P(helix)
	Cost for synthesis		Isoelectric point/P(sheet)
			Volume x Isoelectric Point
			Isoelectric point/P(helix)
			P(helix)/P(sheet)
			Steric hindrance/P(sheet)
			P(helix) x Flexibility
		Set 4	Discrete descriptors
			Number of Oxygen atoms in side chain
			Number of Nitrogen atoms in side chain
			Number of Sulfur atoms in side chain
			Number of H-bond donors and acceptors in side chain

Set 3 extends Set 1 by including precomputed combinations of its descriptors (such as Volume/P(helix)). Set 4 consists of discrete numbers describing the heavy atom compositions of the amino acid side chains. Sets 3 and 4 are experimental and should be used with caution, since many of their descriptors are correlated to those in Sets 1 and 2. Note also that including more descriptors increases the strength of the Bonferroni correction

**Fig. 1:**
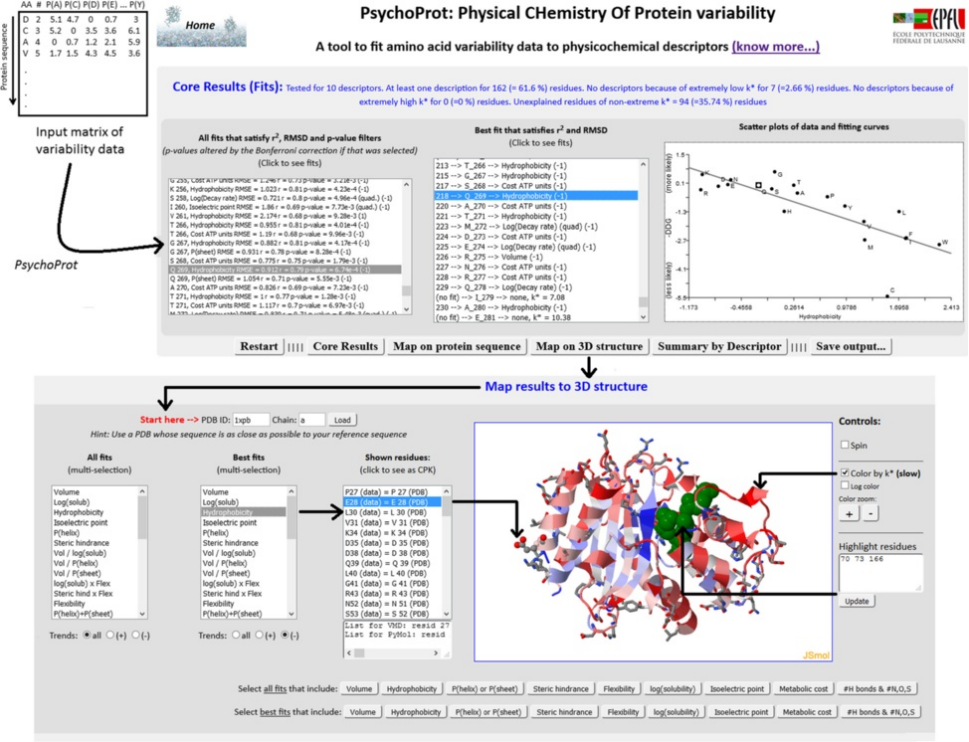
Scheme of the input data and screenshot of *PsychoProt* when displaying the Core Results (top half) and when mapping results on structure (bottom half). In the gray window on top, the list of all fits that satisfy the *p*-value cutoff is on the left, the list with only the best fits for each residue is in the center, and a plot of the currently selected fit is shown on the right. The ribbon of buttons leads to further outputs as exemplified in Additional file 1: Figures S1 and S2. The gray window at the bottom opens when the user clicks “Map on 3D structure” in the ribbon of buttons. Here the user choses first a PDB ID (in this case 1XPB [65]) and the chain corresponding to the protein of interest (top left, labelled “Start here”) and clicks “Load”. The protein backbone is automatically shown as cartoons, here colored according to the tolerance to substitution (k*) which ranges from blue (low tolerance) to white to red (high tolerance). All residues whose best fit was against *hydrophobicity* with a negative trend (*i.e.* favoring hydrophilic amino acids) are shown as sticks (of which Glu28 is also rendered as spheres because it was clicked in the list). Three active site residues are also rendered as green spheres. This collage corresponds to the analysis of amino acid variability in a structure-consistent alignment built from TEM-1’s sequence, and is available as a sample dataset in the website

As a core result *PsychoProt* outputs a list of all the significant fits and another with the best significant fit obtained for each site if any (Fig. 1, top part). Residues for which no fits were obtained or whose tolerance to substitution is extreme (<4 or >16) are flagged in the list of best descriptors. Clicking on the elements of the lists displays plots of the original data points and the corresponding fits on the right. A ribbon of buttons leads to further outputs (bottom part of Fig. 1 and Additional file 1: Figures S1-S2) including a summary of how frequently was each descriptor picked and interactive plots that summarize where the fitted residues map on the protein sequence and structure with the possibility of selectively mapping residues that follow only positive or negative trends on the descriptor. The numerical outputs in text format are also at a button click, useful to compare different datasets, render custom plots and map results on protein structures with external programs.

As a final remark before presenting actual results, we point out that treating each site independently in the fits could possibly offer too many degrees of freedom for finding correlations with amino acid descriptors. This is why we opted to keep the number of descriptors low, and we suggest the use of no more than sets 1 and 2 whose descriptors are low-correlated to each other as well as the use of the Bonferroni correction. Naturally, as with any automated protocol for data analysis, knowledgeable interpretation of the results in the frame of general protein biophysics and considering aspects specific to the subject protein is important.

## Results and discussion

We next exemplify specific applications of *PsychoProt* to real-world datasets, through analyses of amino acid variability in deep-sequence datasets about mutational tolerance in human influenza hemagglutinin [7], in two close variants of human influenza ribonucleoprotein [19] and in a TEM β-lactamase [4], the latter compared to results on a structure-consistent alignment of naturally occurring lactamases; and on structure-consistent alignments of a soluble zinc-dependent β-lactamase and of a tetrameric transmembrane aquaporin.

### Tolerance to substitutions in a viral surface protein that undergoes functional refolding upon interaction with a receptor

**Fig. 2:**
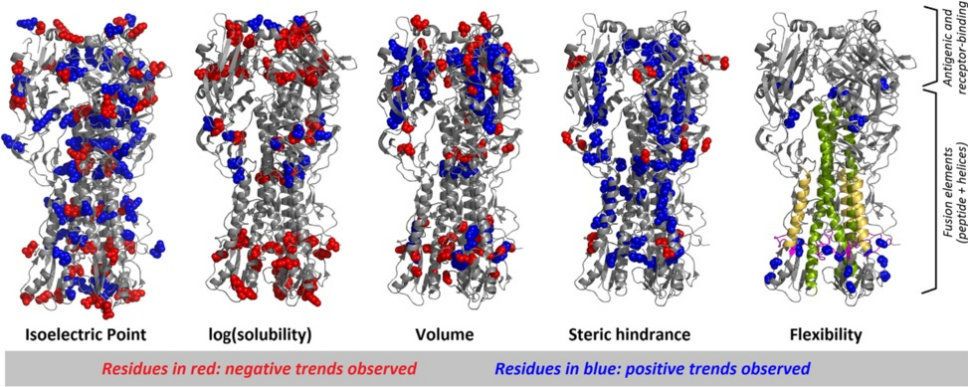
Tolerance to substitutions in human influenza hemagglutinin as quantified by deep-sequencing and explained by *PsychoProt* analyses. Structure mapping of the amino acid descriptors that explain most of the variability observed in a deep-sequencing study of human influenza hemagglutinin (HA). The five most frequent descriptors, out of the eight physicochemical amino acid properties tested, are mapped on the structure of the protein trimer in the metastable prefusion conformation (PDB ID 1RVX). In each picture, residues colored in blue are those for which correlations against the descriptor were positive, while those in red correspond to negative correlations (only positive trends were observed for *flexibility*). In the picture for flexibility, we have also mapped the stalk helices (green), the A-helix (yellow), and the fusion peptide (magenta). These pictures were not rendered in the website but externally with the program PyMOL [66]

Fusion of the human influenza virus to its target cell upon infection is mediated by the hemagglutinin HA glycoprotein. HA is a trimer whose monomers are arranged in a fusion-competent conformation with the fusion-mediating peptides buried inside a trimeric stem (Fig. 2, rightmost structure) [37–39]. Upon interaction with the target receptor in the host, fusion-competent HA undergoes a large conformational change that releases the buried fusion peptides and inserts them into the target membrane, initiating membrane fusion and hence infection. This conformational change is regarded as a “refolding” event originated by relaxation of the complex internal mechanics of the metastable prefusion trimer (corresponding to the structures shown in Fig. 2) [37–39].

Motivated by the ability of the human influenza virus to escape immunity through mutations of its HA protein, Thyagarajan et al. used deep sequencing to evaluate the functional effects of all amino acid substitutions in this protein [7]. They found that HA’s surface residues are more tolerant to substitution than buried residues, as reported for other systems [4, 5]; and that among surface residues there is lower tolerance to substitutions at receptor-binding sites than at antigenic sites, the later related to the virus’ ability to escape the immune response through mutation. Thyagarajan et al. noticed that receptor-binding sites are less solvent exposed than antigenic sites, which could explain their lower tolerance to substitutions, but through a formal test they showed that receptor-binding sites are less tolerant of substitutions than other buried sites.

The initial look at receptor and antigenic sites in the work by Thyagarajan et al. was driven by their low site entropies. These site entropies take values in the range from 1.58 to 4.16 for this dataset, which are not straightforward to interpret. If one provides *PsychoProt* only the amino acid preferences as inputs, it estimates the total tolerance to substitutions (k*) based on equation 2 returning a metric defined in the range from 1 (only one amino acid tolerated) to 20 (all amino acids equally tolerated). The recomputed tolerances (Additional file 1: Table S1) confirm that antigenic sites are indeed highly tolerant of substitutions (with only one site having low tolerance out of 28) and show in a straightforward way that receptor-binding sites are very intolerant of substitutions, with two sites having k* = 10.7 and 16.2 and the rest all ranging between 1 and 2.2.

Moving on to the analysis of amino acid preferences, *PsychoProt* executed with standard settings and considering only physicochemical properties as descriptors (*i.e.* Set 1) yields fits for 168 sites, almost one third of the total number of residues in the dataset (Fig. 2, Additional file 1: Figure S3 and Table 2). Of them, 95 correlate with descriptors directly related to internal contacts, mechanics and flexibility, such as *volume* (37 sites), *steric hindrance* [33], *sheet propensity* [14] and *flexibility* [9]. Other constraints arise from high polarity requirements at the surface of soluble globular proteins and buried hydrophobic residues, *i.e.* surface residues that follow positive correlations with *log(solubility)* or both positive and negative correlations with *isoelectric point*, and buried residues that follow negative correlations against *log(solubility)*. From a functional point of view, it is remarkable that the amino acid preferences for many buried sites follow positive trends against *volume* and *steric hindrance,* meaning that bulky amino acids are preferred, consistent with the idea of a metastable core in the prefusion trimer. Furthermore, several sites following positive correlations against *flexibility* map to the flexible regions that connect the stalk and fusion helices, which undergo large displacements when the fusion peptides refold upon activation (Fig. 2, fusion peptide in magenta and helices in green and yellow). These observations highlight the functional role of internal mechanics and are consistent with the spring-loaded model [40]. Within the framework of such model the metastable prefusion trimer would consist of a tightly packed network of buried residues with flexible hinges localized so as to drive the stress built at the core into swinging movements that release the fusion peptides upon perturbation allowing them to undergo the loop-to-helix transition that embeds them in the target membrane [41].

**Table 2:** Summary of explained and unexplained sites in the four experimental datasets of protein variation, having run *PsychoProt* with only physicochemical descriptors

Protein	HA	RNP 1934	RNP 1968	TEM-1
Source of data	Thyagarajan et al. *eLife* [7]	Doud et al. *Mol Biol Evol* [19]	Doud et al. *Mol Biol Evol* [19]	From Deng et al. [4]
Sites explained by some descriptor	29.8 %	39.8 %	39.6 %	29.7 %
Unexplained of very low tolerance (k* < 4)	14.5 %	15.9 %	7.4 %	38 %
Unexplained of very high tolerance (k* > 16)	23.9 %	5.8 %	19.3 %	0.4 %
Unexplained of not extreme tolerance to substitutions	31.7 %	38.8 %	33.9 %	31.9 %

### Similarities and differences between two variants of a viral nucleic acid-binding nucleoprotein

With the goal of investigating whether amino acid preferences are similar in close protein homologs, Doud et al. interrogated through deep sequencing two strains of the human influenza ribonucleoprotein (RNP variants 1934 and 1968) separated by three decades of evolution and 30 residue substitutions [19]. They found that 14, or possibly up to a maximum of 72, out of the 497 sites analyzed exhibit significantly different amino acid preferences between the two RNP variants, suggesting that site-specific amino acid preferences are quite conserved during short periods of evolution.

Using *PsychoProt* one can further interrogate to what extent the underlying physicochemical, structural and functional constraints remain the same, or vary, between the protein variants of the two strains. Applying *PsychoProt* with standard settings and focusing only on physicochemical properties from Set 1, 198 and 197 sites were fitted to at least one descriptor for variants 1934 and 1968, respectively, *i.e.* ~40 % of their sites (Table 2). 139 of these sites were fitted to at least one descriptor in both variants, accounting for 28 % of the protein length with a similar distribution throughout the sequence as shown in Additional file 1: Figure S4. This means there are 59 sites that were fitted to at least one descriptor in variant 1934 but to none in variant 1968, and 58 sites that were fitted to at least one descriptor in variant 1968 but to none in variant 1934. While some of these differences might reflect true differences in the underlying constraints, they can also be due to noise in the input data large enough to blur trends in one dataset but not in the other, especially given the large size of this protein. Similarly, segments of contiguous sites that were not fitted to any descriptor in any dataset (Additional file 1: Figure S4) likely arise from regions with lower number of measurements and hence higher noise, together with extremely high or low tolerance to substitutions (~20-25 %, as shown in Table 2), intricate dependencies of the amino acid preferences on multiple physicochemical descriptors, and sources of variation arising from effects at transcription, RNA stabilization and degradation, translation, protein degradation, and other levels [42].

**Fig. 3:**
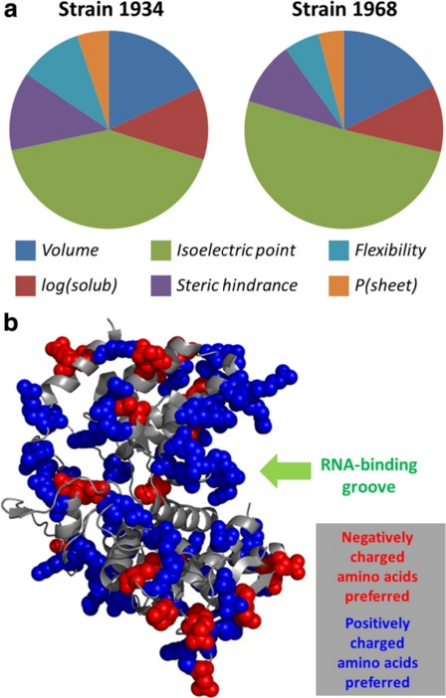
Comparative analyses of deep sequencing data on two closely related HIV RNP variants. **a** Pie charts summarizing how frequently was each amino acid descriptor picked as the best one for sites in RNP from HIV strains 1934 and 1968. **b** Sites shaped by *isoelectric point* with positive (blue) and negative (red) trends in the data for strain 1968 (PDB ID 2IQH [67])

Of the 139 sites that were simultaneously fitted to at least one descriptor in the two RNP variants, 113 (81 %) were fitted to the same descriptor (Additional file 1: Table S2) suggesting largely similar physicochemical constraints acting on both variants and therefore supporting Doud et al.’s main conclusions. But it is the other 26 sites that are more interesting, because they are shaped by different constraints in the two backgrounds, as shown for two examples in Additional file 1: Figure S5 where mutations not only change the encoded amino acids but also seem to change the underlying physicochemical constraints. Notably, though, the overall distribution of counts for each amino acid descriptor selected for best fits are similar in the two variants (Fig. 3a) suggesting that changes in constraints at one site might be compensated by changes in constraints at others.

Another interesting observation is that *isoelectric point* is the most frequent best descriptor in both datasets. Most of the fits against *isoelectric point* show indeed positive trends, consistent with positively charged regions required in the RNA binding groove (Fig. 3b). Two smaller surface patches with several sites that follow negative trends against *isoelectric point*, *i.e.* which favor the presence of negatively charged residues, might be related to the interactions of RNP with other proteins or could serve to compensate the positive charges and avoid an excessively high isoelectric point.

### Natural vs. in vitro variability in TEM lactamases

TEM lactamases are globular, soluble enzymes that confer bacterial resistance towards β-lactams and stand as workhorse systems to study protein evolution in the laboratory through deep sequencing and by analyzing their extensive natural variability [1–4, 43–46]. We used *PsychoProt* to analyze an alignment of natural β-lactamases whose information content is consistent with the structure of the TEM-1 lactamase as shown by the EVFold server [21] (Additional file 1: Figure S6), and compared these results to those on high-resolution deep-sequence data obtained after *in vitro* selection of a saturated library of TEM-1 mutants against ampicillin [4].

**Fig. 4:**
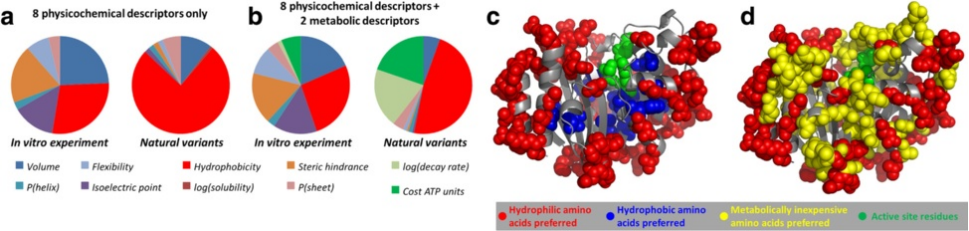
Comparative results on a structure-consistent alignment of natural TEM-like lactamases (“Natural variants”) and on a deep-sequencing experiment on a saturated library of TEM-1 mutants (“In vitro experiment”). Panels A and B compare how frequently each amino acid descriptor was picked as the best in each dataset, considering exclusively physicochemical properties (**a**) or also metabolic descriptors (**b**). Panel (**c**) maps, from the dataset of natural variants, residues shaped by *hydrophobicity* in blue and red spheres (positive and negative trends, respectively, meaning hydrophobic and hydrophilic residues preferred). Green spheres are catalytic residues. Panel (**d**) maps residues shaped negatively by *hydrophobicity* in red and negatively by metabolic descriptors in yellow (meaning metabolically cheap and low-turnover amino acids preferred). Green spheres are catalytic residues (Ser70, Lys73 and Glu166). Pictures in panel C and D were not rendered in the website but externally with PyMOL [66]

Running *PsychoProt* with default settings on physicochemical descriptors only (Set 1) returns fits for roughly 30 % of the sites in the *in vitro* experiment and 44 % of the sites in the alignment. In both cases, the most frequent best descriptor is *hydrophobicity* followed by *volume*; however, the former is by far the most often picked one in the alignment-based data (75.7 % vs. 10.4 % for *volume*) while both are roughly similar in the *in vitro* data (28.2 vs. 24.3 %) (Fig. 4a). This suggests differences in the constraints acting on TEM-1’s sequence in both scenarios. Further interesting, including two metabolic descriptors of the amino acids (Set 2) in the *PsychoProt* run returns a large number of sites where simply metabolically inexpensive amino acids are favored in the alignment data (39.5 %) while this happens on only 7.9 % of the sites in the *in vitro* data (Fig. 4b). Addition of the metabolic descriptors to the analysis does not perturb the number of sites fitted to descriptors of Set 1, such that the findings given above still hold. The results further discussed (and shown in Fig. 4b-d) therefore correspond to those including physicochemical and metabolic descriptors.

In both datasets, most dependencies on *hydrophobicity* are negative (~80 %) and map to the protein surface indicating that hydrophilic residues are favored, while positive dependencies on *hydrophobicity* map to buried locations, as expected for soluble proteins. The negative dependencies on *hydrophobicity* in the alignment-based data are well spread on the surface but quite excluded from a 10 Å sphere around the active site (Fig. 4c), which has an overall low tolerance to substitutions. All sites shaped by metabolic descriptors follow negative trends, such that metabolically inexpensive and low-turnover amino acids are preferred. They mostly map to the protein surface, but contrary to sites shaped by negative trends on *hydrophobicity*, they appear less excluded from the active site (Fig. 4d). Also, they concentrate in patches that could possibly correspond to regions of relatively low importance to the protein’s stability and activity.

In summary, the *in vitro* data seems to capture the constraints on hydrophobicity that act on the natural variants, but it does not capture the effects of amino acid biochemical costs, possibly because the experiments are carried out in rich media with controlled conditions and no limitations on carbon and nitrogen sources. These and other minor differences highlighted by *PsychoProt* indicate important alterations in the constraints that underlie variability in the two datasets. This observation calls for caution when extrapolating conclusions from experimental tolerance-to-substitution maps and fitness landscapes to the natural scenario, adding to a series of potential problems recently discussed [47]. Specifically from this example, sequence constraints related to improving protein solubility and minimizing the use of metabolically expensive amino acids seem to be much more important in nature than *in vitro*, consistent with the previous finding that amino acid metabolism conflicts with protein variation [36].

### Two further examples on the physicochemical factors underlying variation in structure-consistent alignments

**Fig. 5:**
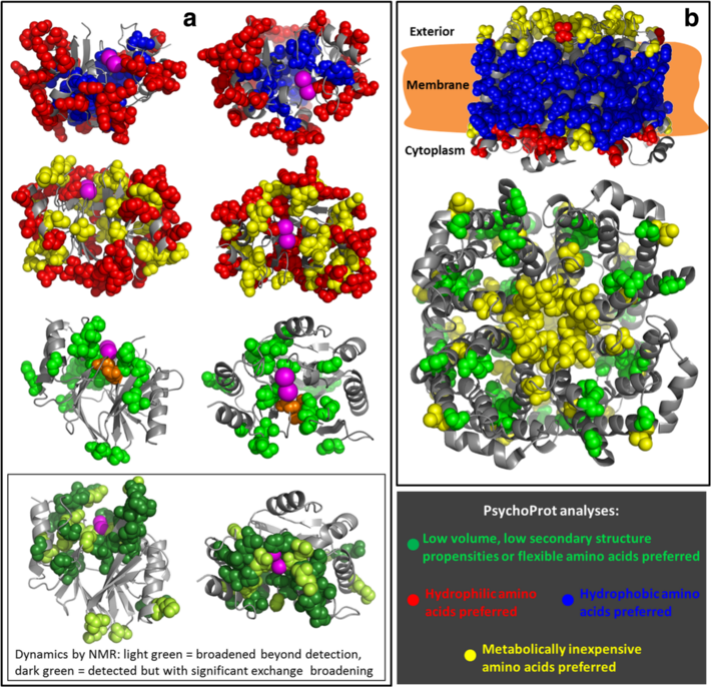
Main results on structure-consistent alignments of natural BcII-like metallo-β-lactamases (**a**) and aquaporins (**b**). In the two panels, residues in blue and red undergo positive and negative constraints on hydrophobicity, respectively; yellow residues follow negative trends against metabolic descriptors (i.e. favoring inexpensive amino acids -no positive trends were observed); and green sites indicate residues were small volumes, small secondary structure propensities or high flexibility are preferred. The inset in panel (**a**) presents NMR data about slow dynamics in extended-spectrum mutants as determined by Gonzalez et al. *Mol Biol Evol* 2016. The structure used in panel (**a**) is PDB ID 1BC2 [68], with the two zinc ions in magenta, while that in panel (**b**) is PDB ID 1SOR [69] copied symmetrically four times to reproduce the biologically relevant tetramer. Each row of pictures in panel (**a**) corresponds to pairs of perpendicular views. The top picture in (**b**) corresponds to a view from “inside” the membrane plane, and the bottom picture is a view from above the membrane. These pictures were not rendered in the website but externally with PyMOL [66]

The large amount of genomic data and novel methods to dissect residue coevolution now allow the computation of protein folds and protein-protein interactions from sequence alignments only [21–27]. These methods enable novel routes for modeling proteins even when no structures for homologues are available; but on top, the alignments from which coevolution is computed can potentially contain other kinds of information as shown above for the TEM-1 lactamase. We briefly report two more interesting analyses carried out with *PsychoProt* on structure-consistent alignments (Fig. 5 and Table 3). We note before delving into these examples, that the term “coevolution” is used here merely to point out that the used alignments include coevolution signatures that are consistent with the contacts observed in protein structures, therefore it is reasonable that they contain rich biophysical information like. (Alignments that are poor in information about residue-residue contacts from coevolution are also expected to be poor informers about the physiochemical traits that shape amino acid preferences.) We further note that the methods used to predict residue-residue contacts from coevolution patterns do not inform about the exact underlying evolutionary processes and phylogenetics.

**Table 3:** Summary of results from the three analyses of structure-consistent protein alignments, using *PsychoProt* with standard parameters and both descriptor sets 1 (physicochemical properties) and 2 (metabolic descriptors)

Protein	TEM-1β-lactamase	BcII metallo-β-lactamase	Aquaporin-0
Soluble/Membrane and oligomerization state	Soluble monomer	Soluble monomer	Transmembrane tetramer
PDB ID	1XPB	1BC2	1SOR
Source of alignment	From EVFold server	From EVFold server	From Ovchinnikov et al. eLife [25]
Sites explained by some descriptor	61.6 %	69.6 %	61.1 %
*Hydrophobicity*	47.5 %	48.7 %	52.6 %
Two metabolic descriptors	39.5 %	27.8 %	17.0 %
*Volume*	5.6 %	11.4 %	15.6 %
Unexplained of very low tolerance (k* < 4)	2.7 %	4.4 %	10.4 %
Unexplained of very high tolerance (k* > 16)	0 %	0.9 %	0 %
Unexplained of not extreme tolerance to substitutions	35.7 %	25.1 %	28.5 %

After having investigated an alignment of TEM lactamases above, we investigate in this section a globular, soluble, zinc-dependent β-lactamase, BcII, widely used as a model system to understand structure, dynamics, catalysis and substrate profiles in metallo-β-lactamases [48] (Fig. 5a). On BcII’s sequence, the EVFold server [21] returns a large alignment whose couplings recapitulate fairly well the protein’s contact map (Additional file 1: Figure S7). *PsychoProt* analysis of this alignment scanning physicochemical and metabolic descriptors (Sets 1 and 2) returns fits for 69.6 % of the residues. As observed for TEM-1 datasets, *hydrophobicity* dominates the trends, again with a large network of surface-exposed sites that favor hydrophilic amino acids and a cluster of buried residues that favor hydrophobic amino acids. Also for BcII, *PsychoProt* reveals several surface residues whose preferences are such that they favor metabolically inexpensive amino acids, and like for TEM-1, they seem to gather and reach into the active site slightly more than negative trends on *hydrophobicity*. We further observe several sites constrained by trends that translate to favoring flexibility, namely either positive trends on *flexibility* itself or negative trends on *volume* and secondary structure propensities (Fig. 5a, bottom). All but two of these residues map to the half of the protein that contains the active site, much like in the pattern of slow-timescale dynamics observed through NMR experiments in BcII variants evolved for an extended substrate profile [49] (inset in Fig. 5a, residues given in Additional file 1: Table S3). This is noteworthy because the family of protein sequences in the alignment includes a large number of zinc-based hydrolases of the metallo-β-lactamase fold with varied substrates. These bioinformatic and experimental findings suggest that at least this family of enzymes exploits roughly half of the fold as a stability reservoir allowing the other half, which contains the active site, to be more fluxional, reminiscent of the idea of fold polarity in enzymes [50]. Also interestingly, the two only residues requiring flexibility far from the active site (Ser49 and Asn137, both also identified as dynamic by NMR in the extended substrate mutant) are far from each other in BcII’s sequence but contact each other in the structure (Fig. 5a).

Finally, we looked at aquaporin-0, a homotetrameric transmembrane protein whose monomers passively transport water across membranes with a number of physiological roles (Fig. 5b). Coevolution analysis on a large alignment of natural sequences with GREMLIN [23] and modeling with Rosetta resulted in a structure essentially identical to the crystallographic one in a recent benchmark by the Baker group [25]. Analysis of that alignment in *PsychoProt* returns fits for 61.1 % of the sites. *Hydrophobicity* accounts for half of the detected trends, but as expected for integral membrane proteins and opposed to what we observed for the soluble proteins, most of these trends are positive (*i.e.* favor hydrophobic amino acids) and correspond to the exposed residues that match the hydrophobic portion of the membrane. Negative trends on *hydrophobicity* gather mostly on the cytoplasmic side of the protein, while metabolic trends (again all negative, favoring inexpensive amino acids) map mostly to the cell exterior. Several sites constrained by small volumes and high flexibility map to the lumen of each monomer, through which water flows.

Interesting conclusions emerge from joint analysis of the three *PsychoProt* runs on structure-consistent alignments (Table 3). In the three cases, the amino acid preferences for 60-70 % of the sites can be explained by physicochemical and metabolic descriptors of the amino acids. Of the ~30-40 % of sites not fitted to any descriptor, ~25 % have very high or low tolerance to substitutions (k* > 16 or k* < 4, respectively), the latter pointing at active sites and interaction surfaces. But the remaining 75 % of sites that were not fitted (~30 % of the total number of residues in the proteins) display intermediate tolerance to substitutions. The lack of fits for these sequence-based examples could be due to multiple reasons. From the protein side, it can be due to very intricate dependencies of the amino acid preferences on physicochemical descriptors or from epistatic effects between sites. Also coevolution effects can impact, such that many pairs of residues vary under the strict constraint of maintaining interactions but since interactions can arise from diverse physicochemical properties (salt bridges, hydrophobic packing, hydrogen bonds, etc.) then no single property is especially preferred. As a more extreme consequence of coevolution effects, the “evolutionary Stokes shift”, *i.e.* the mechanism by which amino acid preferences at certain sites re-adapt to accepted changes at other sites [51], is also expected to blur correlations with amino acid descriptors. (Note that effects related to coevolution apply only to alignment-based data but essentially not to mutational library data because the latter deal largely with single substitutions). Additionally, the lack of fits both for alignment and mutational data can also have contributions from effects at the levels of transcription, RNA stabilization and degradation, translation, protein degradation, and other effects not directly relevant to the folded protein [42]. Notably, the best biophysical models for rates of evolution among sites within proteins (a metric related to, but different than, amino acid preferences) can also explain ~60 % of the observed variance [52].

### Relationship to studies of protein modeling, design and evolution

The kinds of results arising from these studies are strongly related to principles of protein modeling and design, and to those of protein evolution, as highlighted in a recent collection of works at the interface of protein science and molecular evolution [53]. Concerning modeling and design, for example, we learned in the previous section that constraints on hydrophobicity related to the compactness of soluble globular proteins and their surface polarity, or to membrane insertion in transmembrane proteins, are dominant and might therefore be used as additional information for alignment-based modeling of proteins by helping define buried and exposed regions. Constraints from volumes, flexibility and steric hindrance seem more related to functional aspects and could hence be used to approximate the location of active sites and interacting surfaces in protein structures and models, together with metrics for conservation gradients [54].

Regarding the field of protein evolution, careful consideration of the dominant structural, functional and biochemical constraints that shape protein sequences is increasingly recognized as crucial for obtaining models of protein evolution better based on biological, physical and chemical principles [52, 55, 56]. While coarse effects related to protein stability and solvent accessibility have been well demonstrated and are increasingly integrated into evolutionary models [57–59], *PsychoProt* analyses help to find out finer constraints to the identity of the amino acid required/tolerated at each site. We note though that being designed with a structural goal in mind, *PsychoProt* does not deal with the underlying evolutionary processes and phylogenetics, which is the specialty of programs like TreeSAAP [60, 61]. Also, *PsychoProt* does not account for intragenic epistasis, effects of coevolution and the concomitant evolutionary shifts, and constraints that do not act on folded proteins themselves but rather on their translation, folding mechanisms, propensity to degradation, transcription and turnover of their mRNAs, etc. [42] Hopefully, joint consideration of all these elements on top of the site-specific effects detected by *PsychoProt* within a detailed phylogenetic framework will lead in the future to our ultimate understanding of molecular evolution *ab initio* in terms of physical and chemical principles, opening in turn the door to full control during protein modeling and design.

## Methods

### Core algorithm in PsychoProt

For each protein residue with available data, the selected set(s) of amino acid descriptors are screened for correlations by fitting each of them to the observed distribution of amino acids. Fits are retained depending on the *p*-value associated to the probability that they were observed by chance, considering the Bonferroni correction for multiple testing according to the number of descriptors being screened. One picked descriptor is replaced later on by another that also satisfies the *p*-value cutoff only if the improvement is significant according to F-statistics about the decrease of the RMSE between experimental and back-predicted values. By default, linear relationships are fitted, which would lead to monotonically increasing or decreasing patterns. However, the user can choose to search for quadratic dependencies as well, which could help to identify optimal or worst values for a descriptor, and in practice results also in curved yet near-monotonically increasing or decreasing relationships.

The list of descriptors currently available in *PsychoProt* was retrieved from previous works [36, 62, 63] selected such that they have low correlations to each other. When multiple sets of descriptors are selected for screening, *PsychoProt* scans them in the order from Set 1 to 4, to ensure that combinations of descriptors (Set 3) and discrete descriptors (Set 4) are only picked if they truly give a statistically better fit than the simple descriptors.

### PsychoProt’s Inputs and Outputs

The input to *PsychoProt* is a matrix in text format made up of as many rows as residues for which data is available, and at least 22 tab-delimited columns: reference amino acid in one-letter code, residue number, and preferences for all 20 amino acids. If available, an additional column can include a parameter that measures the total tolerance to substitutions (k*) at each site. If that is not provided, *PsychoProt* will estimate it from the amino acid preferences using equation 2. In another optional column the user can include any other data as needed. Sample input datasets are given in the website for a deep-sequencing based-study of TEM-1 lactamase and for a structure-consistent alignment of this protein.

Deep-sequence data must be externally converted to a text-format matrix by the user, for example with a spreadsheet program (tables can be directly pasted to *PsychoProt* or saved to a text file and then loaded). For the analysis of variability in protein alignments, an auxiliary web tool computes a *ΔΔG*_*i,j*_ matrix from the alignment in one step (including the tolerance to substitutions for each residue) and sends it straight to the main analysis tool.

As a main result, displayed under “Core Results (Fits)”, *PsychoProt* delivers two lists; one with all the fits that satisfy the *r*, *RMSE* and p-value limits, and another containing only the best fits for each residue (Fig. 1 of the main paper and Additional file 1: Figure S1). Clicking on items of these lists shows plots of experimental preferences against the selected amino acid descriptor, and the relevant fits (Fig. 1). In these plots, the negative of the amino acid preferences is plotted, such that more favored residues get higher values when the ΔΔG metric from equation 1 is used.

The ribbon of buttons right below the lists and plot offers an easy way to export data in text format (“Save output…”) and toggles between plots. One such plot interactively maps which descriptors were selected at different positions of the sequence (“Map on protein sequence”) with the possibility to show positive, negative or both trends, compare results with the total tolerance to substitutions and with additional data provided in the entry matrix. Another plot summarizes how frequently was each descriptor picked (“Summary by Descriptor”) either singly or grouped, etc. Last, if a PDB structure file is available for the protein or a closely related protein in the Protein Databank, *PsychoProt* features an interactive tool (under “Map on 3D structure”) to map the fits on the 3D structure of the protein, augmented with all the features of JSmol [64] and with an easy way to extract selected residues for external visualization. This tool is intended for on-the-fly mapping of results; however, the user might find it easier to do structural analysis. To facilitate this, a small text box gives prebuilt selections of amino acid residues in formats that the programs VMD and PyMOL understand (to the left of the JSmol widget, under the list of shown residues).

### Data sources for the examples

The example about natural variability in TEM lactamases corresponds exactly to that set by the button “TEM-1 structure-consistent alignment” in the webpage for the main program. This dataset comes from an alignment of proteins retrieved by EVFold’s Jackhammer module using the sequence of the mature portion of the TEM-1 β-lactamase and default parameters for the search [21]. According to EVFold, this alignment contains a large amount of residue coevolution information that is highly consistent with the known structure (Additional file 1: Figure S6), and by design, it has been purged of redundant sequences. The example about *in vitro* variability in TEM lactamases corresponds exactly to that set by the button “TEM-1 deep-sequencing”, and is the experimental dataset by Deng et al. [4] The examples about deep-sequence data for human influenza hemagglutinin and nucleoprotein were produced by the Bloom lab [7, 19]. The antigenic and receptor-binding residues of the hemagglutinin protein were defined as by Thyagarajan et al. [7]. The sequence alignments and coevolution-predicted contact maps for TEM-1 lactamase and BcII metallo-β-lactamase were obtained with the EVCouplings server (http://evfold.org/). The sequence alignment for aquaporin-0 corresponds to the article cited in the main text and was downloaded from the Gremlin website (http://gremlin.bakerlab.org/). Tools used to compare evolutionary couplings and structures are available at http://lucianoabriata.altervista.org/evocoupdisplay/gremlin.html and http://lucianoabriata.altervista.org/evocoupdisplay/evcouplings.htm.

## Abbreviations

BcII, *Bacillus cereus* metallo-βlactamase II; HA, hemagglutinin; RNP, ribonucleoprotein

## Additional file

Additional file 1: Supplementary material available online includes the supplementary Figures S1-S7 and Tables S-S3 mentioned in the article. A Matlab version of PsychoProt’s core functionality is available at the website. (DOCX 931 kb)
