# Erratum to: Detection and sequence/structure mapping of biophysical constraints to protein variation in saturated mutational libraries and protein sequence alignments with a dedicated server

**DOI:** 10.1186/s12859-016-1315-z

**Published:** 2016-10-31

**Authors:** Luciano A. Abriata, Christophe Bovigny, Matteo Dal Peraro

**Affiliations:** 1 Laboratory for Biomolecular Modeling, Institute of Bioengineering, School of Life Sciences, École Polytechnique Fédérale de Lausanne, and Swiss Institute of Bioinformatics, AAB014 Station 19, Lausanne, 1015 Switzerland; 2 Present address: Molecular Modeling Group, Swiss Institute of Bioinformatics, UNIL, Bâtiment Génopode, Lausanne, 1015 Switzerland

## Erratum

After publication of the original article [1] it was brought to our attention that the caption to Figure 3 contained a twice recurring error.

The caption reads: “Comparative analyses of deep sequencing data on two closely related HIV RNP variants. a Pie charts summarizing how frequently was each amino acid descriptor picked as the best one for sites in RNP from HIV strains 1934 and 1968. b Sites shaped by isoelectric point with positive (blue) and negative (red) trends in the data for strain 1968 (PDB ID 2IQH [67])”.

This should instead read: “Comparative analyses of deep sequencing data on two closely related influenza RNP variants. a Pie charts summarizing how frequently was each amino acid descriptor picked as the best one for sites in RNP from influenza strains 1934 and 1968. b Sites shaped by isoelectric point with positive (blue) and negative (red) trends in the data for strain 1968 (PDB ID 2IQH [67])”.
