# Supplementary material for: Detection and sequence/structure mapping of biophysical constraints to protein variation in saturated mutational libraries and protein sequence alignments with a dedicated server
Source: BMC Bioinformatics. 2016 Jun 17;17:242. doi: 10.1186/s12859-016-1124-4 (PMC4912743; doi:10.1186/s12859-016-1124-4)
Supplement: Supplementary file 1 — Supplementary material available online includes the supplementary Figures S1-S7 and Tables S-S3 mentioned in the article. A Matlab version of PsychoProt’s core functionality is available at the website. (DOCX 931 kb) [file 12859_2016_1124_MOESM1_ESM.docx]

Supplementary Material for

Detection and sequence/structure mapping of biophysical constraints to protein variation in saturated mutational libraries and protein sequence alignments with a dedicated server

*Luciano A. Abriata, Christophe Bovigny and Matteo Dal Peraro*

École Polytechnique Fédérale de Lausanne, Switzerland


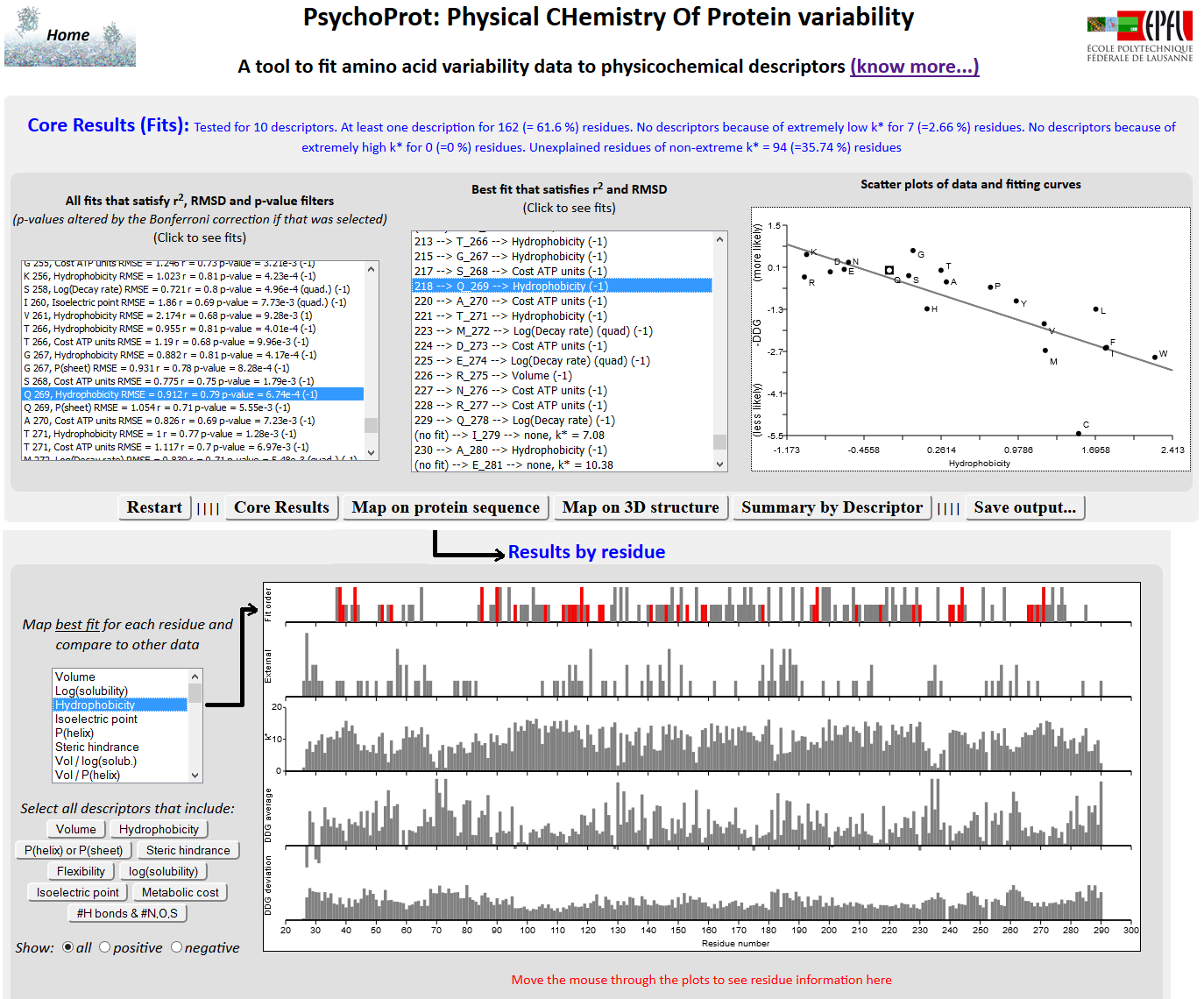


**Figure S1. Example of the interactive mapping of fitted sites on the protein sequence.** Arrows point from clicked buttons and lists to their responses.


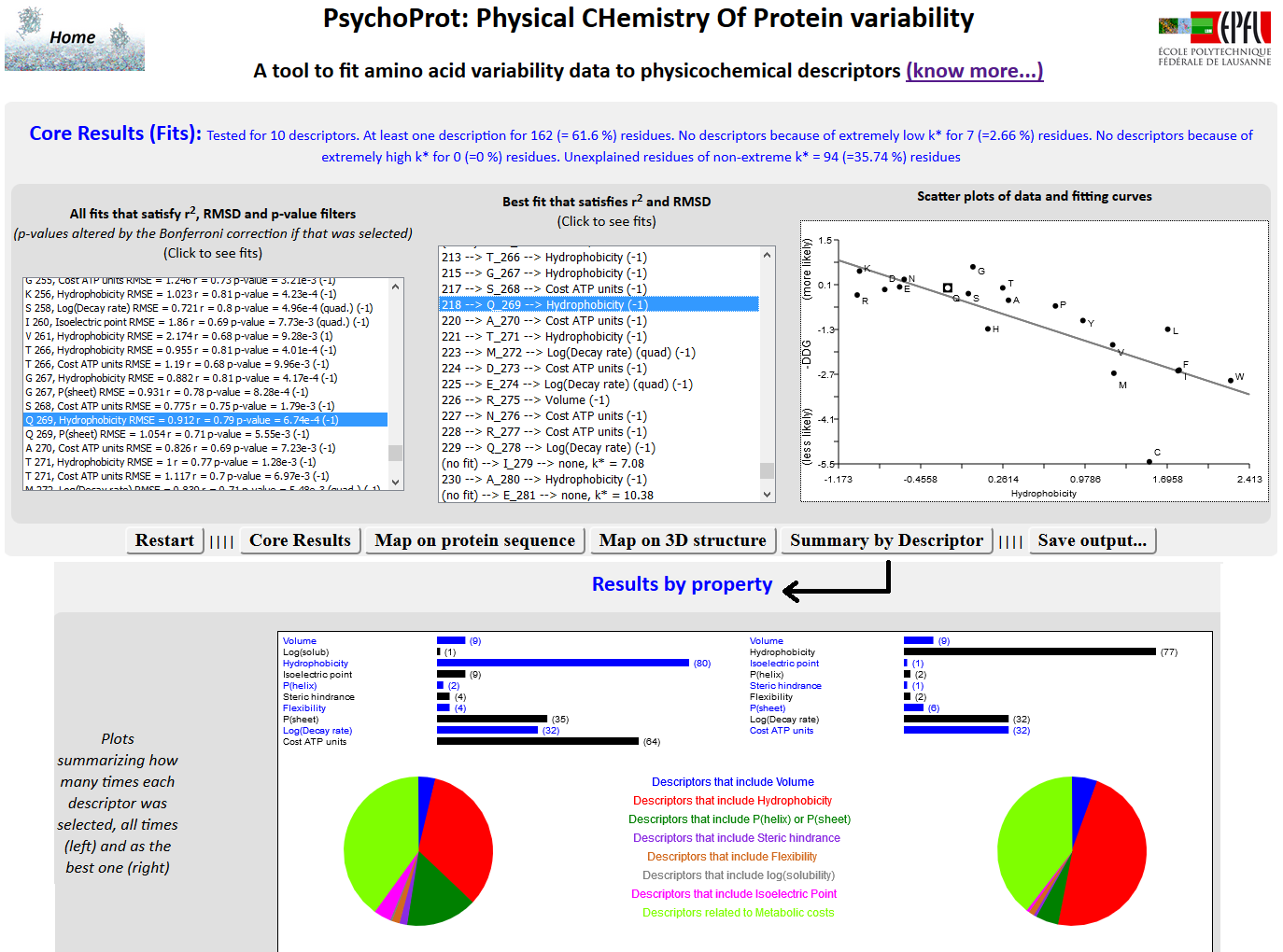


**Figure S2. Example of the interactive plotting of fitted descriptors alone (bars) or grouped by the variables they contain (pie charts).**

**
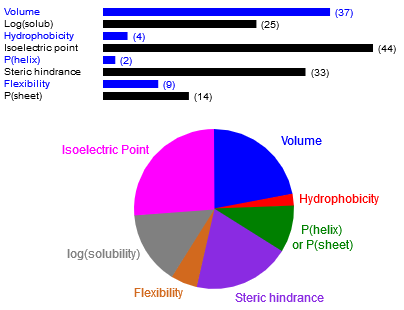
**

**Figure S3. Best fits for the deep-sequence dataset on human influenza hemagglutinin.**


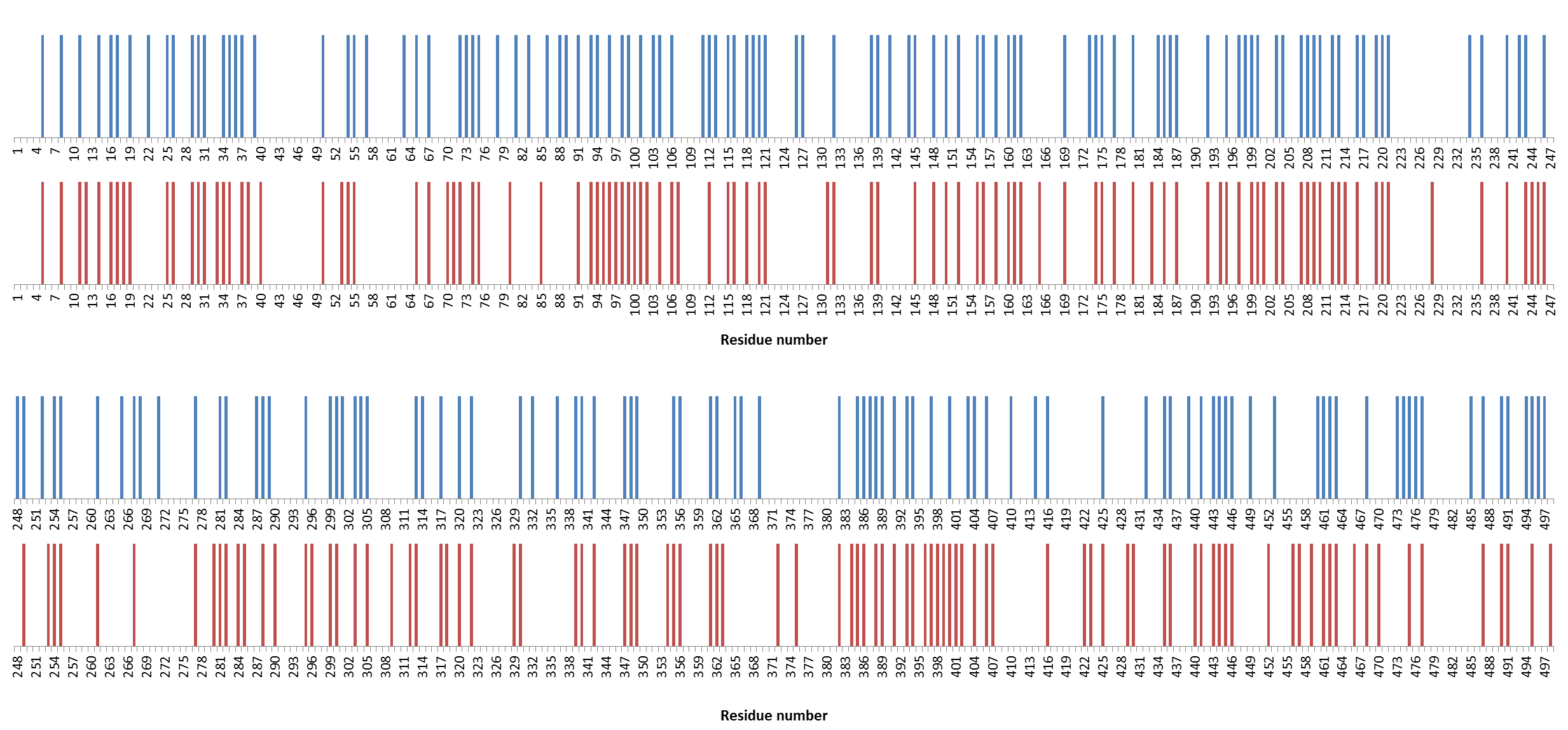


**Figure S4. Sequence mapping of the residues that were fit to some descriptor in the datasets for the two influenza RNP variants**. Strain 1934 is shown in blue; strain 1968 in red.

**
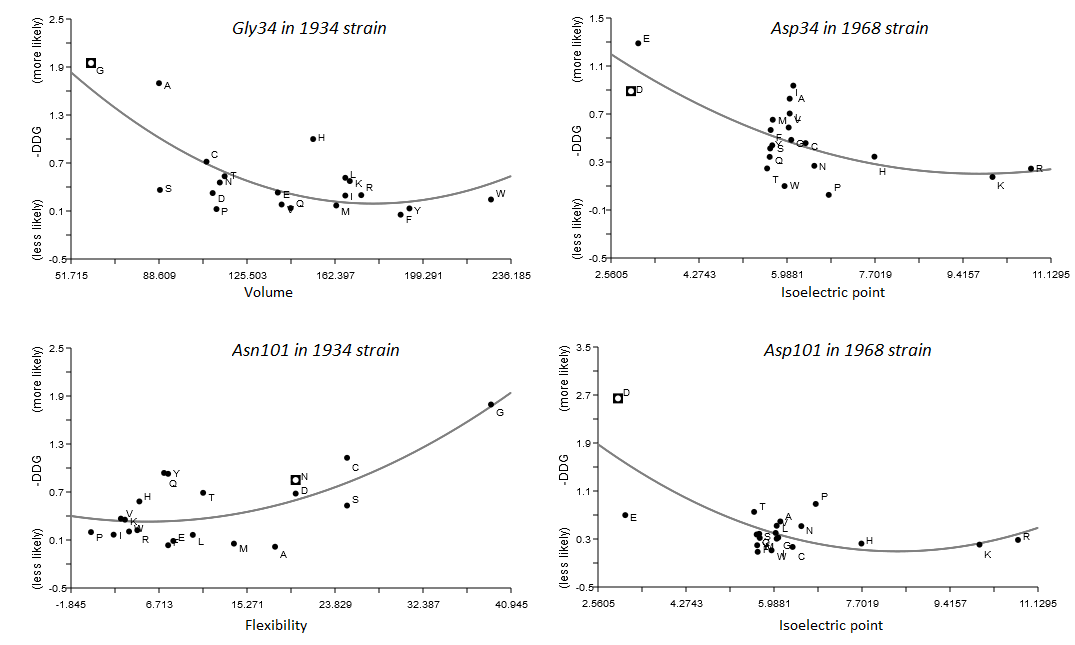
**

**Figure S5. Two examples of sites constrained by different factors in the two RNP variants**.


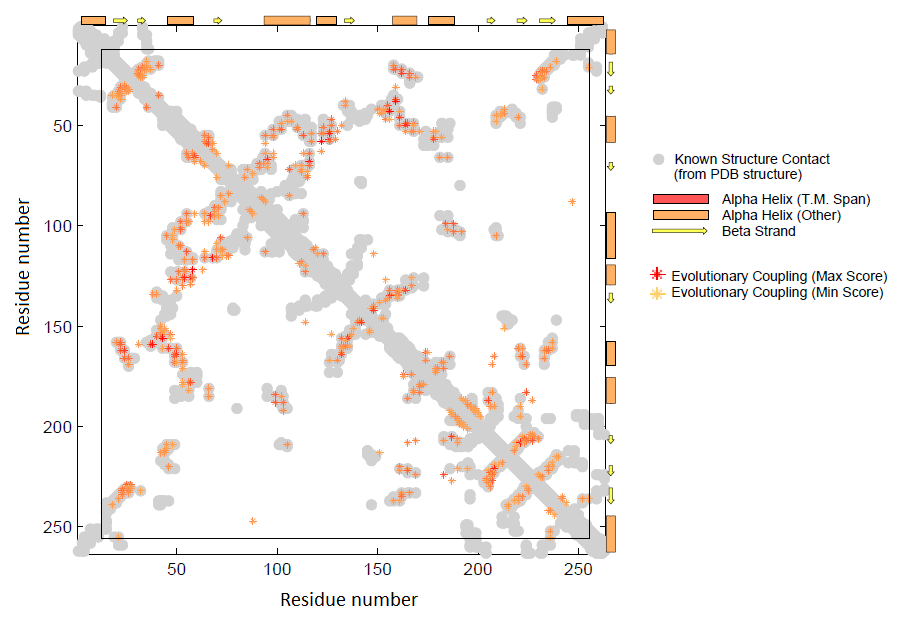


**Figure S6. Couplings predicted by EVFold** from an alignment automatically generated from TEM-1’s sequence (coupling strength increasing from orange to red) compared to a contact map computed from PDB ID 1XPB at 8 Å cutoff (gray).

**
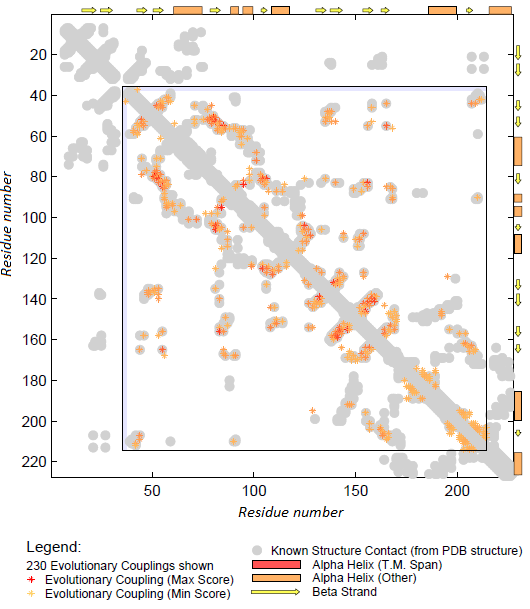
**

**Figure S7. Couplings predicted by EVFold** from an alignment generated from BcII’s sequence (coupling strength increasing from orange to red) compared to a contact map computed from PDB ID 1BC2 at 8 Å cutoff (gray).

**Table S1.** Tolerance to substitutions (in the range from 1 to 20) computed for the influenza hemagglutinin dataset by PsychoProt based on the amino acid preferences.

| **Antigenic sites** | | **Receptor-binding sites** | |
| --- | --- | --- | --- |
| Residue (numbering as in original data) | Total tolerance to substitutions (*k**) recomputed by *PsychoProt* | Residue (numbering as in original data) | Total tolerance to substitutions (*k**) recomputed by *PsychoProt* |
| 87 | 2.8 | 108 | 1.0 |
| 88 | 16.0 | 166 | 1.0 |
| 90 | 18.9 | 196 | 1.2 |
| 91 | 18.6 | 203 | 2.2 |
| 92 | 18.1 | 207 | 1.8 |
| 135 | 12.5 | 208 | 1.0 |
| 141 | 15.1 | 239 | 10.7 |
| 151 | 9.4 | 241 | 1.1 |
| 156 | 16.1 | 253 | 16.2 |
| 158 | 16.4 |  |  |
| 169 | 18.7 |  |  |
| 171 | 17.4 |  |  |
| 172 | 18.1 |  |  |
| 173 | 19.2 |  |  |
| 175 | 19.1 |  |  |
| 176 | 19.1 |  |  |
| 178 | 18.1 |  |  |
| 179 | 15.5 |  |  |
| 180 | 19.0 |  |  |
| 182 | 18.6 |  |  |
| 186 | 16.8 |  |  |
| 205 | 13.6 |  |  |
| 206 | 17.7 |  |  |
| 209 | 16.9 |  |  |
| 211 | 16.3 |  |  |
| 220 | 15.4 |  |  |
| 237 | 15.1 |  |  |
| 238 | 10.0 |  |  |

**Table S2.** Comparison of fits retrieved for strains 1934 and 1968 of human influenza RNP. When no fit was achieved, the total tolerance to substitutions k* is shown.

| **Residue (S1934)** | **Best descriptor** | **Residue (S1968)** | **Best descriptor** |
| --- | --- | --- | --- |
| G 5 | Flexibility | G 5 | Volume |
| R 8 | Isoelectric point | R 8 | Isoelectric point |
| E 11 | Isoelectric point | E 11 | Isoelectric point |
| Q 12 | none, k* = 15.04 | Q 12 | Isoelectric point |
| E 14 | Isoelectric point | E 14 | Hydrophobicity |
| D 16 | Isoelectric point | D 16 | Isoelectric point |
| G 17 | Flexibility | G 17 | Volume |
| E 18 | none, k* = 14.56 | E 18 | Isoelectric point |
| R 19 | Isoelectric point | R 19 | Isoelectric point |
| A 22 | Volume | A 22 | none, k* = 14.39 |
| I 25 | Steric hindrance | I 25 | Steric hindrance |
| R 26 | Isoelectric point | R 26 | Isoelectric point |
| V 29 | P(sheet) | V 29 | Steric hindrance |
| G 30 | Volume | G 30 | Steric hindrance |
| K 31 | Isoelectric point | K 31 | Isoelectric point |
| I 33 | none, k* = 7.57 | I 33 | Steric hindrance |
| G 34 | Volume | D 34 | Isoelectric point |
| G 35 | Volume | G 35 | Flexibility |
| I 36 | Steric hindrance | I 36 | none, k* = 14.28 |
| G 37 | Volume | G 37 | Volume |
| R 38 | none, k* = 12.95 | R 38 | Isoelectric point |
| F 39 | Volume | F 39 | none, k* = 16.86 |
| Y 40 | none, k* = 7.19 | Y 40 | Log(solub) |
| S 50 | Flexibility | S 50 | Volume |
| E 53 | none, k* = 1.47 | E 53 | Isoelectric point |
| G 54 | Flexibility | G 54 | Volume |
| R 55 | Isoelectric point | R 55 | Isoelectric point |
| I 57 | Steric hindrance | I 57 | none, k* = 13.85 |
| I 63 | Steric hindrance | I 63 | none, k* = 16.46 |
| R 65 | Isoelectric point | R 65 | Isoelectric point |
| V 67 | P(sheet) | V 67 | Steric hindrance |
| A 70 | none, k* = 5.34 | A 70 | Volume |
| F 71 | none, k* = 5.13 | F 71 | Volume |
| D 72 | Isoelectric point | D 72 | Isoelectric point |
| E 73 | P(helix) | E 73 | none, k* = 18.82 |
| R 74 | Isoelectric point | R 74 | Isoelectric point |
| R 75 | Isoelectric point | R 75 | Isoelectric point |
| Y 78 | Log(solub) | Y 78 | none, k* = 11.73 |
| E 80 | none, k* = 10.1 | E 80 | Isoelectric point |
| E 81 | Log(solub) | E 81 | none, k* = 15.01 |
| P 83 | Log(solub) | P 83 | none, k* = 15.81 |
| A 85 | none, k* = 14.83 | A 85 | Hydrophobicity |
| G 86 | Steric hindrance | G 86 | none, k* = 16.72 |
| D 88 | Isoelectric point | D 88 | none, k* = 3.5 |
| P 89 | P(helix) | P 89 | none, k* = 17.47 |
| K 91 | Isoelectric point | K 91 | Isoelectric point |
| G 93 | Volume | G 93 | Volume |
| G 94 | Volume | G 94 | Volume |
| P 95 | none, k* = 8.69 | P 95 | Log(solub) |
| I 96 | Steric hindrance | I 96 | Steric hindrance |
| Y 97 | none, k* = 12.91 | Y 97 | Log(solub) |
| R 98 | Isoelectric point | K 98 | Isoelectric point |
| R 99 | Isoelectric point | R 99 | Isoelectric point |
| V 100 | none, k* = 17.64 | V 100 | Hydrophobicity |
| N 101 | Flexibility | D 101 | Isoelectric point |
| G 102 | none, k* = 15.12 | R 102 | Isoelectric point |
| K 103 | Isoelectric point | K 103 | none, k* = 17.61 |
| W 104 | Volume | W 104 | Volume |
| R 106 | Isoelectric point | R 106 | Isoelectric point |
| E 107 | none, k* = 9.36 | E 107 | Isoelectric point |
| Y 111 | Log(solub) | Y 111 | none, k* = 15.73 |
| D 112 | Isoelectric point | D 112 | Isoelectric point |
| K 113 | Isoelectric point | K 113 | none, k* = 2.04 |
| E 115 | Isoelectric point | E 115 | Isoelectric point |
| I 116 | Steric hindrance | I 116 | Steric hindrance |
| R 118 | Isoelectric point | R 118 | Isoelectric point |
| I 119 | Steric hindrance | I 119 | none, k* = 15.2 |
| W 120 | Volume | W 120 | Volume |
| R 121 | Isoelectric point | R 121 | Isoelectric point |
| G 126 | Volume | G 126 | none, k* = 17.64 |
| D 127 | Log(solub) | D 127 | none, k* = 19.35 |
| A 131 | none, k* = 16.35 | A 131 | Log(solub) |
| G 132 | Flexibility | G 132 | Flexibility |
| I 138 | Steric hindrance | I 138 | Steric hindrance |
| W 139 | Volume | W 139 | Volume |
| S 141 | Volume | S 141 | none, k* = 6.99 |
| N 144 | Flexibility | N 144 | none, k* = 7.03 |
| D 145 | Isoelectric point | D 145 | Isoelectric point |
| Y 148 | Log(solub) | Y 148 | Log(solub) |
| R 150 | Isoelectric point | R 150 | Isoelectric point |
| R 152 | Isoelectric point | R 152 | Isoelectric point |
| V 155 | P(sheet) | V 155 | P(sheet) |
| R 156 | Isoelectric point | R 156 | Isoelectric point |
| G 158 | Flexibility | G 158 | Flexibility |
| D 160 | Isoelectric point | D 160 | Isoelectric point |
| P 161 | Log(solub) | P 161 | Log(solub) |
| R 162 | Isoelectric point | R 162 | Isoelectric point |
| S 165 | none, k* = 3.12 | S 165 | Flexibility |
| G 169 | Flexibility | G 169 | Volume |
| P 173 | Log(solub) | P 173 | none, k* = 10.12 |
| R 174 | Isoelectric point | R 174 | Isoelectric point |
| R 175 | Isoelectric point | R 175 | Isoelectric point |
| G 177 | Flexibility | G 177 | Volume |
| G 180 | Flexibility | G 180 | Volume |
| V 183 | none, k* = 11.9 | V 183 | P(sheet) |
| K 184 | Isoelectric point | K 184 | none, k* = 2.45 |
| G 185 | Volume | G 185 | Volume |
| V 186 | P(sheet) | V 186 | none, k* = 16.16 |
| G 187 | Flexibility | G 187 | Volume |
| E 192 | Isoelectric point | E 192 | Isoelectric point |
| V 194 | none, k* = 13.59 | I 194 | Steric hindrance |
| R 195 | Isoelectric point | R 195 | Isoelectric point |
| I 197 | Steric hindrance | I 197 | Steric hindrance |
| K 198 | Isoelectric point | K 198 | none, k* = 4.45 |
| R 199 | Isoelectric point | R 199 | Isoelectric point |
| G 200 | Flexibility | G 200 | Volume |
| I 201 | none, k* = 14.91 | I 201 | Steric hindrance |
| D 203 | Isoelectric point | D 203 | Isoelectric point |
| R 204 | Isoelectric point | R 204 | Isoelectric point |
| W 207 | Volume | W 207 | Volume |
| R 208 | Isoelectric point | R 208 | Isoelectric point |
| G 209 | Flexibility | G 209 | Flexibility |
| E 210 | Isoelectric point | E 210 | Isoelectric point |
| G 212 | Volume | G 212 | Volume |
| R 213 | Isoelectric point | R 213 | Isoelectric point |
| K 214 | none, k* = 12.68 | K 214 | Isoelectric point |
| R 216 | Isoelectric point | R 216 | Isoelectric point |
| I 217 | Steric hindrance | S 217 | none, k* = 18.38 |
| Y 219 | Log(solub) | Y 219 | Log(solub) |
| E 220 | Isoelectric point | E 220 | Isoelectric point |
| R 221 | Isoelectric point | R 221 | Isoelectric point |
| G 228 | none, k* = 7.15 | G 228 | Flexibility |
| A 234 | Volume | A 234 | none, k* = 15.16 |
| K 236 | Isoelectric point | R 236 | Isoelectric point |
| D 240 | Isoelectric point | D 240 | Isoelectric point |
| V 242 | P(sheet) | V 242 | none, k* = 10.5 |
| R 243 | Isoelectric point | R 243 | Isoelectric point |
| E 244 | none, k* = 1.56 | E 244 | Isoelectric point |
| S 245 | none, k* = 4.92 | S 245 | Volume |
| R 246 | Isoelectric point | R 246 | Isoelectric point |
| P 248 | Log(solub) | P 248 | none, k* = 15.39 |
| G 249 | Volume | G 249 | Volume |
| E 252 | Isoelectric point | E 252 | none, k* = 2.67 |
| F 253 | none, k* = 5.84 | I 253 | Steric hindrance |
| E 254 | Isoelectric point | E 254 | Isoelectric point |
| D 255 | Isoelectric point | D 255 | Isoelectric point |
| R 261 | Isoelectric point | R 261 | Isoelectric point |
| I 265 | Steric hindrance | I 265 | none, k* = 15.63 |
| R 267 | Isoelectric point | R 267 | Isoelectric point |
| G 268 | Log(solub) | G 268 | none, k* = 14.75 |
| A 271 | Flexibility | A 271 | none, k* = 11.19 |
| P 277 | Log(solub) | P 277 | Log(solub) |
| V 280 | none, k* = 12.82 | V 280 | Steric hindrance |
| Y 281 | Log(solub) | Y 281 | Log(solub) |
| G 282 | Volume | G 282 | Flexibility |
| A 284 | none, k* = 10.46 | A 284 | Isoelectric point |
| V 285 | none, k* = 17.65 | V 285 | P(sheet) |
| S 287 | Isoelectric point | S 287 | none, k* = 18.46 |
| G 288 | Volume | G 288 | Steric hindrance |
| Y 289 | P(sheet) | Y 289 | none, k* = 16.66 |
| D 290 | none, k* = 14.24 | D 290 | Isoelectric point |
| G 295 | Volume | G 295 | Volume |
| Y 296 | none, k* = 13.3 | Y 296 | Log(solub) |
| V 299 | P(sheet) | V 299 | P(sheet) |
| G 300 | Volume | G 300 | Flexibility |
| I 301 | Steric hindrance | I 301 | none, k* = 17.92 |
| P 303 | Log(solub) | P 303 | Log(solub) |
| F 304 | Volume | F 304 | none, k* = 14.62 |
| R 305 | Isoelectric point | K 305 | Isoelectric point |
| N 309 | none, k* = 18.49 | N 309 | Isoelectric point |
| V 312 | none, k* = 13.29 | V 312 | P(sheet) |
| Y 313 | Log(solub) | Y 313 | Log(solub) |
| S 314 | Flexibility | S 314 | none, k* = 12.85 |
| R 317 | Isoelectric point | R 317 | Isoelectric point |
| P 318 | none, k* = 18.34 | P 318 | Log(solub) |
| E 320 | Isoelectric point | E 320 | Isoelectric point |
| P 322 | Log(solub) | P 322 | Log(solub) |
| V 329 | none, k* = 11.62 | V 329 | P(sheet) |
| W 330 | Volume | W 330 | Volume |
| A 332 | Volume | A 332 | none, k* = 5.24 |
| A 336 | Volume | A 336 | none, k* = 10.28 |
| E 339 | Isoelectric point | E 339 | Isoelectric point |
| D 340 | Isoelectric point | D 340 | Isoelectric point |
| R 342 | Isoelectric point | R 342 | Isoelectric point |
| I 347 | Steric hindrance | I 347 | Hydrophobicity |
| K 348 | Isoelectric point | R 348 | Isoelectric point |
| G 349 | Flexibility | G 349 | Volume |
| P 354 | none, k* = 12.93 | P 354 | Log(solub) |
| R 355 | Isoelectric point | R 355 | Isoelectric point |
| G 356 | Flexibility | G 356 | Volume |
| R 361 | Isoelectric point | R 361 | Isoelectric point |
| G 362 | Volume | G 362 | Volume |
| V 363 | none, k* = 9.53 | V 363 | P(sheet) |
| I 365 | Steric hindrance | I 365 | none, k* = 9.25 |
| A 366 | Volume | A 366 | none, k* = 3.88 |
| E 369 | Isoelectric point | E 369 | none, k* = 5.74 |
| E 372 | none, k* = 6.49 | D 372 | Isoelectric point |
| E 375 | none, k* = 13.22 | E 375 | Isoelectric point |
| R 382 | Isoelectric point | R 382 | Isoelectric point |
| R 384 | none, k* = 11.87 | R 384 | Isoelectric point |
| Y 385 | Log(solub) | Y 385 | Log(solub) |
| W 386 | Volume | W 386 | Volume |
| A 387 | Volume | A 387 | none, k* = 4.64 |
| I 388 | Steric hindrance | I 388 | Steric hindrance |
| R 389 | Isoelectric point | R 389 | Isoelectric point |
| R 391 | Isoelectric point | R 391 | Isoelectric point |
| G 393 | Steric hindrance | G 393 | Flexibility |
| G 394 | Volume | G 394 | Volume |
| T 396 | none, k* = 13.64 | T 396 | Isoelectric point |
| N 397 | Isoelectric point | N 397 | Isoelectric point |
| Q 398 | none, k* = 14.48 | Q 398 | Isoelectric point |
| Q 399 | none, k* = 17.74 | Q 399 | Isoelectric point |
| R 400 | Isoelectric point | R 400 | Isoelectric point |
| A 401 | none, k* = 14.12 | A 401 | Isoelectric point |
| S 402 | none, k* = 14.62 | S 402 | Isoelectric point |
| A 403 | Flexibility | A 403 | none, k* = 15.8 |
| G 404 | Flexibility | G 404 | Volume |
| I 406 | Steric hindrance | I 406 | Steric hindrance |
| S 407 | none, k* = 1.82 | S 407 | Flexibility |
| P 410 | Log(solub) | P 410 | none, k* = 3.97 |
| V 414 | Steric hindrance | V 414 | none, k* = 13.94 |
| R 416 | Isoelectric point | R 416 | Isoelectric point |
| R 422 | none, k* = 14.45 | K 422 | Isoelectric point |
| T 423 | none, k* = 9.63 | P 423 | Isoelectric point |
| I 425 | Steric hindrance | I 425 | P(sheet) |
| F 429 | none, k* = 1.63 | F 429 | Log(solub) |
| N 430 | none, k* = 9.99 | T 430 | Log(solub) |
| N 432 | P(helix) | N 432 | none, k* = 19.08 |
| G 435 | Volume | G 435 | Volume |
| R 436 | Isoelectric point | R 436 | Isoelectric point |
| D 439 | Isoelectric point | D 439 | none, k* = 6.63 |
| M 440 | none, k* = 5.3 | M 440 | Log(solub) |
| R 441 | Isoelectric point | R 441 | Isoelectric point |
| E 443 | Isoelectric point | E 443 | Isoelectric point |
| I 444 | Steric hindrance | I 444 | Steric hindrance |
| I 445 | Steric hindrance | I 445 | Steric hindrance |
| R 446 | Isoelectric point | R 446 | Isoelectric point |
| E 449 | Isoelectric point | E 449 | none, k* = 12.72 |
| R 452 | none, k* = 16.27 | K 452 | Isoelectric point |
| P 453 | Steric hindrance | P 453 | none, k* = 18.51 |
| V 456 | none, k* = 15.51 | M 456 | Isoelectric point |
| S 457 | none, k* = 8.75 | S 457 | Flexibility |
| Q 459 | none, k* = 18.06 | Q 459 | Isoelectric point |
| G 460 | Steric hindrance | G 460 | none, k* = 15.74 |
| R 461 | Isoelectric point | R 461 | Isoelectric point |
| G 462 | Volume | G 462 | Volume |
| V 463 | P(sheet) | V 463 | Steric hindrance |
| L 466 | none, k* = 8.59 | L 466 | Volume |
| D 468 | Isoelectric point | D 468 | Isoelectric point |
| K 470 | none, k* = 1.55 | R 470 | Isoelectric point |
| S 473 | P(sheet) | N 473 | none, k* = 17.46 |
| P 474 | Log(solub) | P 474 | none, k* = 10.03 |
| I 475 | Steric hindrance | I 475 | Steric hindrance |
| V 476 | P(sheet) | V 476 | none, k* = 9.37 |
| P 477 | Log(solub) | P 477 | Log(solub) |
| G 485 | Volume | G 485 | none, k* = 13.13 |
| Y 487 | Log(solub) | Y 487 | Log(solub) |
| G 490 | Volume | G 490 | Volume |
| D 491 | Isoelectric point | D 491 | Isoelectric point |
| E 494 | Isoelectric point | E 494 | none, k* = 8.79 |
| E 495 | Isoelectric point | E 495 | Isoelectric point |
| Y 496 | Log(solub) | Y 496 | none, k* = 12.37 |
| D 497 | Log(solub) | D 497 | none, k* = 17.95 |
| N 498 | none, k* = 13.54 | N 498 | Isoelectric point |

**Table S3.** Details for data shown in the inset in Figure 5A. Residues flagged as dynamic in μs–ms timescales by NMR experiments on a double mutant of BcII with extended substrate spectrum (Gonzalez et al *Mol. Biol. Evol.* 2016) and residues whose amino acid distributions are shaped by preferences by high flexibility, low volumes or low secondary structure propensities (this work). Gray shading highlights matching residues.

| NMR data for enhanced-spectrum double mutant (Gonzalez et al *Mol Biol Evol* 2016) | PsychoProt results on structure-consistent alignment of BcII-like sequences (This work) | NMR data (cont.) | PsychoProt results (cont.) |
| --- | --- | --- | --- |
|  | V8 |  | G136 |
| K23 |  | N137 | N137 |
| E30 | E30 |  | Y144 |
| L31 |  | G146 |  |
| G32 |  |  | D152 |
| S33 |  |  | N153 |
| N35 |  | V155 |  |
| G36 |  | L164 |  |
| E37 |  |  | G167 |
| A38 |  | C168 | C168 |
|  | P40 | L169 |  |
| S41 | S41 | V170 |  |
| N42 |  | K171 |  |
| G43 |  | S172 | S172 |
| S49 | S49 |  | T173 |
| S58 |  |  | S174 |
| W59 |  | L178 |  |
| K73 |  | G179 | G179 |
| R78 |  | N180 |  |
|  | T85 | V181 |  |
| H88 |  | A182 |  |
| A89 |  | W189 |  |
| D90 |  |  | K198 |
| R91 |  | V206 |  |
|  | G93 | G209 |  |
|  | T108 | G211 |  |
|  | V130 | G214 |  |
|  |  | G217 | G217 |
|  |  | L219 |  |
|  |  |  |  |
